# Symptoms and recovery among adult outpatients with and without COVID‐19 at 11 healthcare facilities—July 2020, United States

**DOI:** 10.1111/irv.12832

**Published:** 2021-01-06

**Authors:** Kiva A. Fisher, Samantha M. Olson, Mark W. Tenforde, Wesley H. Self, Michael Wu, Christopher J. Lindsell, Nathan I. Shapiro, D. Clark Files, Kevin W. Gibbs, Heidi L. Erickson, Matthew E. Prekker, Jay S. Steingrub, Matthew C. Exline, Daniel J. Henning, Jennifer G. Wilson, Samuel M. Brown, Ithan D. Peltan, Todd W. Rice, David N. Hager, Adit A. Ginde, H. Keipp Talbot, Jonathan D. Casey, Carlos G. Grijalva, Brendan Flannery, Manish M. Patel, Leora R. Feldstein, Kimberly W. Hart, Robert McClellan, Hsi‐nien Tan, Adrienne Baughman, Nora A. Hennesy, Brittany Grear, Kristin Mlynarczyk, Luc Marzano, Zuwena Plata, Alexis Caplan, Constance E. Ogokeh, Emily R. Smith, Sara S. Kim, Eric P. Griggs, Bridget Richards, Sonya Robinson, Kaylee Kim, Ahmed M. Kassem, Courtney N. Sciarratta, Paula L. Marcet

**Affiliations:** ^1^ CDC COVID‐19 Response Team Centers for Disease Control and Prevention (CDC) Atlanta GA USA; ^2^ Epidemic Intelligence Service CDC Atlanta GA USA; ^3^ Influenza Vaccine Effectiveness in the Critically Ill (IVY) Network USA; ^4^ Vanderbilt University Medical Center Nashville TN USA; ^5^ Beth Israel Deaconess Medical Center Boston MA USA; ^6^ Wake Forest University Baptist Medical Center Winston‐Salem NC USA; ^7^ Hennepin County Medical Center Minneapolis MN USA; ^8^ Baystate Medical Center Springfield MA USA; ^9^ Ohio State University Wexner Medical Center Columbus OH USA; ^10^ University of Washington Medical Center Seattle WA USA; ^11^ Stanford University Medical Center Palo Alto CA USA; ^12^ Intermountain Healthcare Salt Lake City UT USA; ^13^ Johns Hopkins Hospital Baltimore MD USA; ^14^ University of Colorado School of Medicine Aurora CO USA

**Keywords:** anosmia, convalescence, COVID‐19, quality of life, recovery, SARS‐CoV‐2, symptoms duration

## Abstract

**Background:**

Symptoms of mild COVID‐19 illness are non‐specific and may persist for prolonged periods. Effects on quality of life of persistent poor physical or mental health associated with COVID‐19 are not well understood.

**Methods:**

Adults aged ≥18 years with laboratory‐confirmed COVID‐19 and matched control patients who tested negative for SARS‐CoV‐2 infection at outpatient facilities associated with 11 medical centers in the United States were interviewed to assess symptoms, illness duration, and health‐related quality of life. Duration of symptoms, health‐related quality of life measures, and days of poor physical health by symptoms experienced during illness were compared between case patients and controls using Wilcoxon rank‐sum tests. Symptoms associated with COVID‐19 case status were evaluated by multivariable logistic regression.

**Results:**

Among 320 participants included, 157 were COVID‐19 cases and 163 were SARS‐CoV‐2 negative controls. Loss of taste or smell was reported by 63% of cases and 6% of controls and was strongly associated with COVID‐19 in logistic regression models (adjusted odds ratio [aOR] = 32.4; 95% confidence interval [CI], 12.6‐83.1). COVID‐19 cases were more likely than controls to have experienced fever, body aches, weakness, or fatigue during illness, and to report ≥1 persistent symptom more than 14 days after symptom onset (50% vs 32%, *P* < .001). Cases reported significantly more days of poor physical health during the past 14 days than controls (*P* < .01).

**Conclusions:**

Differentiating COVID‐19 from other acute illnesses will require widespread diagnostic testing, especially during influenza seasons. Persistent COVID‐19‐related symptoms may negatively affect quality of life, even among those initially presenting with mild illness.

## 1 INTRODUCTION

The coronavirus disease 2019 (COVID‐19) pandemic has caused significant morbidity and mortality worldwide. COVID‐19 disease severity ranges from asymptomatic illness to death.^1^ Symptoms associated with SARS‐CoV‐2 infection are non‐specific, making it difficult to distinguish COVID‐19 from other diseases. Recent reports suggest that recovery from COVID‐19 can be prolonged, even when initial symptoms are mild.^2^, ^3^ Effects of prolonged recovery on health‐related quality of life, including physical health, mental health, and return to usual activities, have received little attention for COVID‐19.^4^

Descriptions of symptoms during the acute phase of COVID‐19 have mainly relied on case series^5^, ^6^; few studies have reported symptom profiles and return to baseline health using a comparison group without COVID‐19. The objective of this investigation was to compare symptom prevalence and recovery among adults with and without COVID‐19 who were tested at outpatient health facilities for SARS‐CoV‐2 infection during July 2020.

## 2 METHODS

### 2.1 Design

A matched case‐control study was performed among a random sample of adults (≥18 years) tested for SARS‐CoV‐2 at outpatient facilities during July 1‐29, 2020. A COVID‐19 case was laboratory‐confirmed for SARS‐CoV‐2 by reverse transcriptase polymerase chain reaction (RT‐PCR). Control participants (“controls”) were defined as SARS‐CoV‐2 RT‐PCR–negative results in patients who were symptomatic and tested at the same health facilities. Potentially eligible patients were randomly selected in a ratio of 2 controls for each case patient from lists of adults who were tested at outpatient facilities associated with 11 academic medical centers in the Influenza Vaccine Effectiveness in the Critically Ill (IVY) Network.^7^ Once confirmed case patients were randomly selected, 2 controls were matched to each case patient by sex, age, study site, and location of testing. Participants were eligible if, at the time of telephone interview, they reported the reason for testing as feeling unwell and no previous SARS‐CoV‐2 test. This project was reviewed by CDC and was conducted consistent with applicable federal law and CDC policy. The project was determined to be non‐research public health surveillance by Vanderbilt University Medical Center and CDC.^1^

### 2.2 Measures

Participants were contacted by telephone beginning 14‐26 days after patient's test date. Participant demographics and self‐reported underlying chronic medical conditions were entered into REDCap® software.^8^ Participants were asked how many days they felt unwell or had symptoms prior to getting tested. For each reported symptom or combination of symptoms (eg, nausea or vomiting or loss of appetite; body aches or weakness or fatigue; runny nose or congestion or sore throat; and loss of taste or smell), patients were asked if they experienced the symptom during illness, as well as if the symptom was still present at the time of the interview. Symptom question response options were binary (yes/no). For all symptoms, participants were asked to recall the number of days they experienced the symptom. Questions about convalescence, or time to recovery, were asked at the time of the interview.

### 2.3 Health‐related quality of life

Participants' health‐related quality of life at the time of interview was assessed using modified questions from the Healthy Days Measures developed by the Centers for Disease Control and Prevention.^9^ Because eligible persons were contacted >14 days after first SARS‐CoV‐2 test, participants were asked to think about their physical and mental health during the past 14 days, and report the number of days they experienced poor physical health, mental health, or limitations performing usual activities due to poor health. Responses ranged from 0 to 14 days. All participants, including COVID‐19 cases and controls were asked four questions from the Healthy Days Core Module and five questions from the Healthy Days Symptoms Module.

### 2.4 Analysis

Differences between case patients and controls were compared using chi‐square tests and analysis of variance to assess demographic characteristics, underlying chronic condition and symptom prevalence. Symptom duration and health‐related quality of life measures were compared between case patients and controls using Wilcoxon rank‐sum tests. Because of a large number of unmatched pairs, due in part to the strict inclusion criteria (ie, symptomatic at the time of first test), unconditional logistic regression accounting for site‐level clustering was used to assess symptom differences between case patients and controls, adjusting for race/ethnicity, sex, age, and presence of ≥1 underlying chronic medical condition.^10^ Adjusted odds ratios (aOR) and 95% confidence intervals (CI) are reported. Significance levels were set at *P* < .05. Analyses were performed using SAS version 9.4 software (SAS Institute).

## 3 RESULTS

After randomization and matching, a total of 1827 participants were identified for inclusion (615 case patients and 1212 controls). Of those, 163 adults declined to participate. Of 802 adults successfully contacted who agreed to participate (295 cases and 507 controls), 470 were not eligible for the investigation (ie, reported no symptoms, had multiple tests, or both). Among 332 eligible participants, 12 interviews were excluded because participants did not answer symptom questions. The final sample included 320 participants, 157 COVID‐19 case patients and 163 controls. Participants reported a median of 3 days (IQR, 2‐4) of symptoms before SARS‐CoV‐2 test; interviews were conducted 15‐52 days (median, 22 days) after illness onset. The participants were 53% female; the median age for both case patients and controls was 40 years (cases IQR, 28‐53 years vs controls IQR, 30‐53 years). Compared with controls, case patients were less likely to report being White, non‐Hispanic, (60% vs 77%; *P* = .005), less likely to have completed college (51% vs 67%; *P* < .001), and less likely to report ≥1 underlying chronic medical condition (48% vs 61%; *P* = .02).

**FIGURE 1:**
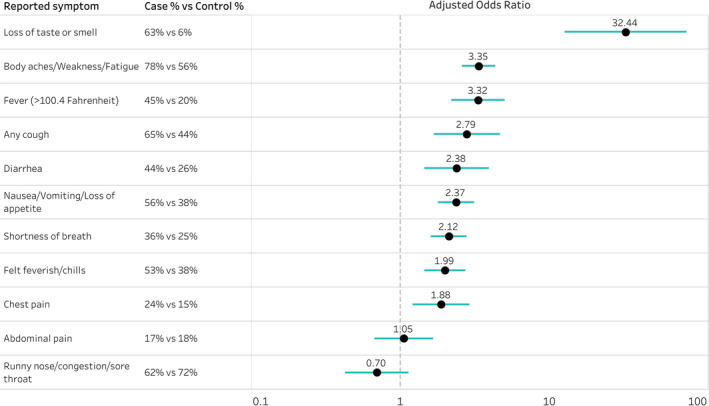
Adjusted^a^ odds ratios comparing odds of reported symptoms among COVID‐19 cases^b^ compared with controls^c^, July 2020. ^a^Adjusted for race/ethnicity, sex, age, and presence of ≥1 underlying chronic medical condition (at least one of the following underlying chronic medical conditions: cardiac condition, hypertension, asthma, chronic obstructive pulmonary disease (COPD), immunodeficiency, psychiatric condition, diabetes, or obesity). ^b^A COVID‐19 case was laboratory‐confirmed for SARS‐CoV‐2 by reverse transcriptase polymerase chain reaction (RT‐PCR). ^c^Control participants tested negative for SARS‐CoV‐2 infection by RT‐PCR at the same health facilities. Potentially eligible patients were randomly selected in a ratio of 2 controls for each case patient from lists of adults who were tested at outpatient clinics associated with 11 academic medical centers participating in the Influenza Vaccine Effectiveness in the Critically Ill (IVY) Network

COVID‐19 cases reported higher prevalence than controls for 9 of 11 categories of symptoms assessed (Table 1). In logistic regression models adjusted for age, sex, race/ethnicity and presence of ≥1 underlying chronic medical condition, the strongest association with COVID‐19 was observed for reported loss of taste or smell (63% of cases vs 6% of controls, aOR = 32.4; 95% CI, 12.6‐83.1), while fever (>100.4° Fahrenheit; 45% vs 20%, aOR = 3.3; 95% CI, 2.2‐5.0) and body aches, weakness, or fatigue (78% vs 56%, aOR = 3.3; 95% CI, 2.6‐4.3) were also more likely to be reported among case patients than controls (Figure 1).

**TABLE 1:** Frequency and duration of reported symptoms experienced during acute illness among laboratory‐confirmed COVID‐19 cases compared with controls who tested negative for SARS‐CoV‐2 infection, July 2020

Symptom^a^	Cases (SARS‐CoV‐2 positive), N = 157		Controls (SARS‐CoV‐2 negative), N = 163		*P*‐value^b^
	No. responses (%)	No. days, median (IQR)	No. responses (%)	No. days, median (IQR)	
Days of illness	121 (77%)	12 (8‐15)	138 (85%)	6 (4‐9)	<.01
Loss of taste or smell	98 (63%)	7 (4‐14)	9 (6%)	5 (3‐9)	.30
Body aches/weakness/fatigue	121 (78%)	10 (5‐16)	91 (56%)	5 (3‐10)	<.01
Fever (>100.4 Fahrenheit)	69 (45%)	3 (2‐5)	33 (20%)	2 (1‐4)	.16
Any cough	102 (65%)	7 (4‐14)	72 (44%)	6 (2‐8)	.11
Diarrhea	69 (44%)	4 (2‐7)	42 (26%)	3 (2‐5)	.30
Nausea/vomiting/loss of appetite	88 (56%)	5 (3‐10)	61 (38%)	3 (2‐5)	<.01
Shortness of breath	57 (37%)	7 (5‐14)	41 (25%)	4 (3‐8)	.01
Felt feverish/chills	81 (53%)	3 (2‐5)	61 (38%)	3 (2‐5)	.84
Chest pain	37 (24%)	2 (1‐5)	24 (15%)	2 (1‐4)	.45
Abdominal pain	26 (17%)	3 (1‐5)	29 (18%)	4 (2‐5)	.43
Runny nose/congestion/sore throat	98 (62%)	6 (3‐10)	117 (72%)	5 (3‐8)	.61

^a^ Participants who reported experiencing symptom during illness were asked to recall symptom duration, in days. Responses were missing or unknown for reported loss of taste or smell (n = 3), body aches/weakness/fatigue (n = 3), fever (n = 5), diarrhea (n = 1), nausea/vomiting/loss of appetite (n = 2), shortness of breath (n = 1), chills (n = 4), abdominal pain (n = 1), and runny nose (n = 1). Symptom duration was missing or unknown for reported loss of taste or smell (n = 1), body aches/weakness/fatigue (n = 2), cough (n = 5), diarrhea (n = 2), nausea/vomiting/loss of appetite (n = 3), shortness of breath (n = 1), chills (n = 2), abdominal pain (n = 2), chest pain (n = 4), and runny nose (n = 5).

^b^ *P*‐value from Wilcoxon rank‐sum test for difference in number of days of symptoms reported among COVID‐19 cases and controls.

Case patients had a longer recovery period from their illness compared with controls. Among participants who reported each symptom or combination of symptoms, body aches or weakness or fatigue was reported for longer duration among COVID‐19 cases than controls (median, 10 days [IQR, 5‐15] vs 5 days [IQR, 3‐10]; *P* < .001). At the time of interview, 50% of cases and 32% of controls reported ≥1 persistent symptom (*P* < .001); 35% of cases and 13% of controls reported persistent body aches, weakness or fatigue (*P* < .001).

Referring to the 14 days before interview, case patients reported a median of 5 days (IQR, 0‐9) of poor physical health and four days (IQR, 0‐7) of limited activity due to poor health, vs 0 days (IQR, 0‐3; *P* < .01) of poor physical health among controls (Table 2). Poor mental health was less commonly reported and did not differ between cases and controls. COVID‐19 cases reported feeling healthy and full of energy a median of 5 (IQR, 1‐12) of the past 14 days vs 8 of 14 days (IQR, 3‐12) among controls (*P* = .04). Among both case and control participants, days of poor physical health were significantly correlated with symptoms experienced during illness and with symptom duration. Reported shortness of breath, chest pain, or abdominal pain at any time during illness were each associated with the number of days in the past 14 days during which participants reported poor physical health (*P* ≤ .01); cases experiencing these symptoms reported a median of 8 or more days of poor physical health. Among participants with ≥1 persistent symptom at the time of interview, case patients reported a median of 8 days (IQR, 4‐13) of poor physical health, vs 2 days (IQR, 0‐5) among controls (*P* < .01).

**TABLE 2:** Number of days that COVID‐19 cases and controls without COVID‐19 reported impacts on physical or mental health, or limitations with usual activities

Healthy days measure^a^	Cases (SARS‐CoV‐2 positive) (n = 157)		Controls (SARS‐CoV‐2 negative) (n = 163)		*P*‐value^b^
	No. responses	No. days, median (IQR)	No. responses	No. days, median (IQR)	
Not good physical health	156	5 (0‐9)	162	0 (0‐3)	<.01
Not good mental health	155	0 (0‐7)	160	1 (0‐4)	.91
Activity limitation	155	4 (0‐7)	162	0 (0‐3)	<.01
Pain	154	0 (0‐6)	161	0 (0‐1)	<.01
Sad, depressed, blue	155	0 (0‐4)	161	0 (0‐2)	.26
Anxious	154	1 (0‐7)	160	1 (0‐6)	.87
Sleepless	153	2 (0‐6)	162	3 (0‐7)	.07
Full of energy	155	5 (1‐12)	159	8 (3‐12)	.04
**Symptom experienced during illness**	**No. responses**	**No. days of poor physical health, median (IQR)**	**No. responses**	**No. days of poor physical health, median (IQR)**	***P*‐value** ^b^
Fever (>100.4 Fahrenheit)	69	7 (1‐11)	33	1 (0‐3)	<.01
Felt feverish/chills	80	6 (1‐10)	61	1 (0‐3)	<.01
Shortness of breath	56	8 (5‐13)	41	1 (0‐6)	<.01
Any cough	101	6 (2‐10)	71	0 (0‐3)	<.01
Chest pain	36	8 (3‐13)	24	2 (0‐9)	.01
Abdominal pain	25	10 (7‐14)	29	2 (0‐7)	<.01
Nausea/vomiting/loss of appetite	87	7 (3‐11)	61	1 (0‐5)	<.01
Diarrhea	68	7 (5‐13)	42	1 (0‐3)	<.01
Body aches/weakness/fatigue	120	7 (3‐10)	91	1 (0‐4)	<.01
Runny nose/congestion/sore throat	98	6 (0‐11)	117	0 (0‐3)	<.01
Loss of taste or smell	97	6 (2‐10)	9	2 (0‐4)	.10
Persistent symptom(s) during interview	78	8 (4‐13)	51	2 (0‐5)	<.01

^a^ All participants were asked four questions adapted from Healthy Days Core Module and five questions from the Healthy Days Symptoms Module: “Would you say that in general your health is excellent, very good, good, fair, or poor?” (data not shown); “Now thinking about your physical health, which includes physical illness and injury, for how many days during the past 14 days was your physical health not good?” (Not good physical health); “Now thinking about your mental health, which includes stress, depression, and problems with emotions, for how many days during the past 14 days was your mental health not good?” (Not good mental health); “During the past 14 days, for about how many days did poor physical or mental health keep you from doing your usual activities, such as self‐care, work, or recreation?” (Activity limitation); “During the past 14 days, for about how many days did pain make it hard for you to do your usual activities, such as self‐care, work, or recreation?” (Pain); “During the past 14 days, for about how many days have you felt sad, blue, or depressed?” (Sad, depressed, blue); “During the past 14 days, for about how many days have you felt worried, tense, or anxious?” (Anxious); “During the past 14 days, for about how many days have you felt you did not get enough rest or sleep?” (Sleepless); “During the past 30 days, for about how many days have you felt very healthy and full of energy?” (Full of energy).

^b^ *P*‐value from Wilcoxon rank‐sum test for difference in number of days of symptoms and the difference in number of days of poor physical health reported among COVID‐19 cases and controls without COVID‐19.

## 4 DISCUSSION

Our findings show that adults with laboratory‐confirmed COVID‐19 reported a broad range of symptoms and prolonged recovery compared with a matched comparison group of symptomatic adults who tested negative for SARS‐CoV‐2 infection. Compared with controls, case patients had over 30 times the odds of experiencing loss of taste or smell during acute illness. This finding is consistent with other reports that provide evidence for anosmia and dysgeusia associated with COVID‐19 positivity.^11^, ^12^ In the current study, body aches, weakness, and fatigue were reported more frequently among case patients compared with controls. Studies have reported fatigue as a prominent clinical manifestation of COVID‐19.^13^ These findings highlight challenges differentiating COVID‐19 from common causes of acute illness among patients presenting to outpatient facilities. While report of loss of taste or smell may be highly predictive of SARS‐CoV‐2 infection, widespread testing will be needed to identify COVID‐19 patients with mild illness, especially during co‐circulation with influenza and other respiratory viruses.

COVID‐19 also has prolonged effects on both physical and mental health during recovery, even among patients with mild symptoms. Recognition of a post‐viral syndrome characterized by persistent symptoms is growing.^14^, ^15^, ^16^ Symptoms associated with this syndrome include fatigue, chills and sweats, body aches, difficulty concentrating (or “brain fog”), difficulty breathing or shortness of breath, and delayed return to pre‐COVID‐19 health. In addition, some patients have experienced anxiety and sleep disturbances.^14^, ^15^ However, we did not observe a difference in these measures between cases and controls. Examination of health‐related quality of life measures during recovery from acute illness was a novel aspect of this study. COVID‐19 cases experienced significantly more days of poor physical health due to poor health than controls. In contrast, there were no differences in reported days of poor mental health in the past 14 days. The low prevalence of poor mental health was surprising given the documented effects of pandemic mitigation measures including isolation on mental health in the general population.^17^ These results add to reports of negative effects of persistent symptoms on quality of life over longer periods.^2^, ^4^ Finally, symptoms experienced during acute illness may be related to return to usual health; not only symptom duration but their effects on usual activities likely contribute to feelings of poor physical and mental health. These findings suggest that even outpatient illness associated with SARS‐CoV‐2 infection can have significant effects on physical and mental health.

This study was subject to several limitations. First, participants were aware of their test result (ie, negative or positive) at the time of the interview. Knowing one's test outcome as well as recall bias could have impacted responses to both symptom reporting and return to baseline health. Further, although 1:2 case‐control matching by age, sex, and testing site was pre‐specified in the study design, unconditional logistic regression, including matching procedure variables, was used instead of conditional logistic regression accounting for matching due to exclusion of negative control patients who declined to participate, were tested when asymptomatic, or had previously been tested for SARS‐CoV‐2 infection. In addition, case status was subject to misclassification due to imperfect sensitivity and specificity of RT‐PCR assays used at the testing sites. All participants were tested at outpatient facilities and reported duration of symptoms experienced during illness; however, the extent of symptom severity was not measured. Finally, CDC's Healthy Days Measures have not been validated to assess health‐related quality of life during the convalescent phase of acute illness. To better align with the timing of interviews in this study, validated Healthy Days Modules were modified to refer to the past 14 days (instead of the past 30 days) and results may not be comparable to those of surveys such as the Behavior Risk Factor Surveillance Survey (http://www.cdc.gov/brfss).

Clinicians should emphasize that people with COVID‐19–like symptoms need to isolate at the time of symptom onset and seek testing early. Alongside other mitigation efforts, timely isolation of people with symptoms can reduce community transmission of SARS‐CoV‐2. Monitoring and tracking differences among symptomatic adult outpatients with respiratory illness can inform testing guidelines and better characterize SARS‐CoV‐2 infection and COVID‐19, which will have important implications during the influenza season in the United States.

## DISCLOSURES

Carlos G. Grijalva reports grants from Campbell Alliance, the National Institutes of Health, the Food and Drug Administration, the Agency for Health Care Research and Quality and Sanofi‐Pasteur, and consultation fees from Pfizer, Merck, and Sanofi‐Pasteur. Christopher J. Lindsell reports grants from National Institutes of Health and the Department of Defense and other support from Marcus Foundation, Endpoint Health, Entegrion, bioMerieux, and Bioscape Digital, outside the submitted work. Nathan I. Shapiro reports grants from the National Institutes of Health, Rapid Pathogen Screening, Inflammatix, and Baxter, outside the submitted work. Daniel J. Henning reports personal fees from CytoVale and grants from Baxter, outside the submitted work. Samuel M. Brown reports grants from National Institutes of Health, Department of Defense, Intermountain Research and Medical Foundation, and Janssen and consulting fees paid to his employer from Faron and Sedana, outside the submitted work. Ithan D. Peltan reports grants from the National Institutes of Health, Asahi Kasei Pharma, Immunexpress Inc, Janssen Pharmaceuticals, and Regeneron, outside the submitted work. Todd W. Rice reports personal fees from Cumberland Pharmaceuticals, Inc, Cytovale, Inc, and Avisa, LLC, outside the submitted work. Adit A. Ginde reports grants from the National Institutes of Health and Department of Defense, outside the submitted work. H. Keipp Talbot reports serving on the Data Safety Monitoring Board for Seqirus. No other potential conflicts of interest were disclosed.

## AUTHOR CONTRIBUTION

**Kiva Fisher:** Conceptualization (equal); Formal analysis (lead); Investigation (lead); Methodology (equal); Project administration (lead); Visualization (equal); Writing‐original draft (lead). **Samantha M Olson:** Formal analysis (equal); Visualization (equal); Writing‐original draft (supporting); Writing‐review & editing (equal). **Mark W Tenforde:** Conceptualization (equal); Formal analysis (supporting); Methodology (equal); Writing‐original draft (supporting); Writing‐review & editing (equal). **Wesley H Self:** Conceptualization (equal); Funding acquisition (lead); Methodology (equal); Resources (lead); Supervision (equal); Writing‐original draft (supporting); Writing‐review & editing (supporting). **Michael Wu:** Investigation (supporting); Visualization (equal); Writing‐review & editing (supporting). **Christopher J Lindsell:** Project administration (equal); Software (equal); Writing‐review & editing (supporting). **Nathan I Shapiro:** Resources (lead); Writing‐review & editing (supporting). **D. Clarke Files:** Resources (lead); Writing‐review & editing (supporting). **Kevin Gibbs:** Resources (lead); Writing‐review & editing (supporting). **Heidi Erickson:** Resources (lead); Writing‐review & editing (supporting). **Matthew E Prekker:** Resources (lead); Writing‐review & editing (supporting). **Jay Steingrub:** Resources (lead); Writing‐review & editing (supporting). **Matthew Exline:** Resources (lead); Writing‐review & editing (supporting). **Daniel Henning:** Resources (lead); Writing‐review & editing (supporting). **Jennifer Wilson:** Resources (lead); Writing‐review & editing (supporting). **Samuel Brown:** Resources (lead); Writing‐review & editing (supporting). **Ithan Peltan:** Resources (lead); Writing‐review & editing (supporting). **Todd W Rice:** Resources (lead); Writing‐review & editing (supporting). **David N Hager:** Resources (lead); Writing‐review & editing (supporting). **Adit Ginde:** Resources (lead); Writing‐review & editing (supporting). **Keipp Talbot:** Resources (lead); Writing‐review & editing (supporting). **Jonathan D Casey:** Resources (lead); Writing‐review & editing (supporting). **Carlos G Grijalva:** Resources (lead); Writing‐review & editing (supporting). **Brendan Flannery:** Conceptualization (equal); Formal analysis (equal); Methodology (equal); Supervision (lead); Writing‐original draft (supporting); Writing‐review & editing (equal). **Manish M Patel:** Conceptualization (lead); Methodology (lead); Supervision (equal); Writing‐original draft (supporting); Writing‐review & editing (equal). **Leora R Feldstein:** Conceptualization (equal); Methodology (equal); Project administration (equal); Supervision (equal); Writing‐original draft (supporting); Writing‐review & editing (equal).

## DISCLAIMER

The findings and conclusions in this report are those of the authors and do not necessarily represent the official position of the Centers for Disease Control and Prevention.

### PEER REVIEW

The peer review history for this article is available at https://publons.com/publon/10.1111/irv.12832.

## Data Availability

A de‐identified dataset for this study may be requested from CDC [www.cdc.gov/info].

## Footnotes

^1^ Project IRB number 200 591 from Vanderbilt University, IRB Health Sciences Committee #3.

## References

[1] Wu Z , McGoogan JM . Characteristics of and important lessons from the coronavirus disease 2019 (COVID‐19) outbreak in China: summary of a report of 72314 cases from the Chinese Center for Disease Control and Prevention. JAMA. 2020;323:1239‐1242. 32091533 10.1001/jama.2020.2648

[2] Carfi A , Bernabei R , Landi F ; Gemelli Against C‐P‐ACSG . Persistent symptoms in patients after acute COVID‐19. JAMA. 2020;324:603‐605. 32644129 10.1001/jama.2020.12603 PMC7349096

[3] Tenforde MW , Kim SS , Lindsell CJ , et al. Symptom duration and risk factors for delayed return to usual health among outpatients with COVID‐19 in a multistate health care systems network ‐ United States, March‐June 2020. MMWR Morb Mortal Wkly Rep. 2020;69:993‐998. 32730238 10.15585/mmwr.mm6930e1 PMC7392393

[4] Temperoni C , Barchiesi F . Clinical characteristics, management and health‐related quality of life in young adults with COVID‐19. Research Square. 2020. 10.1186/s12879-021-05841-1 PMC7848882 33522907

[5] Burke RM , Killerby ME , Newton S , et al. Symptom profiles of a convenience sample of patients with COVID‐19 ‐ United States, January‐April 2020. MMWR Morb Mortal Wkly Rep. 2020;69:904‐908. 32673296 10.15585/mmwr.mm6928a2 PMC7366851

[6] Tenforde MW , Billig Rose E , Lindsell CJ , et al. Characteristics of adult outpatients and inpatients with COVID‐19 ‐ 11 academic medical centers, United States, March‐May 2020. MMWR Morb Mortal Wkly Rep. 2020;69:841‐846. 32614810 10.15585/mmwr.mm6926e3 PMC7332092

[7] Fisher KA , Tenforde MW , Feldstein LR , et al. Community and close contact exposures associated with COVID‐19 among symptomatic adults >/=18 years in 11 outpatient health care facilities ‐ United States, July 2020. MMWR Morb Mortal Wkly Rep. 2020;69:1258‐1264. 32915165 10.15585/mmwr.mm6936a5 PMC7499837

[8] Harris PA , Taylor R , Minor BL , et al. The REDCap consortium: building an international community of software platform partners. J Biomed Inform. 2019;95:103208. 31078660 10.1016/j.jbi.2019.103208 PMC7254481

[9] Centers for Disease Control and Prevention (CDC) . Measuring Healthy Days. Population Assessment of Health‐Related Quality of Life. Atlanta, GA: Centers for Disease Control and Prevention; 2000.

[10] Tenforde MW , Kim SS , Lindsell CJ , et al. Symptom duration and risk factors for delayed return to usual health among outpatients with COVID‐19 in a multistate health care systems network—United States, March–June 2020. MMWR Morb Mortal Wkly Rep. 2020;69:993‐998. 32730238 10.15585/mmwr.mm6930e1 PMC7392393

[11] Dawson P , Rabold EM , Laws RL , et al. Loss of taste and smell as distinguishing symptoms of COVID‐19. Clin Infect Dis. 2020. 10.1093/cid/ciaa799 PMC7337666 32562541

[12] Lechien JR , Chiesa‐Estomba CM , Hans S , Barillari MR , Jouffe L , Saussez S . Loss of smell and taste in 2013 European patients with mild to moderate COVID‐19. Ann Intern Med. 2020;173:672‐675. 32449883 10.7326/M20-2428 PMC7505100

[13] Tu H , Tu S , Gao S , Shao A , Sheng J . Current epidemiological and clinical features of COVID‐19; a global perspective from China. J Infect. 2020;81:1‐9. 32315723 10.1016/j.jinf.2020.04.011 PMC7166041

[14] Davido B , Seang S , Tubiana R , de Truchis P . Post‐COVID‐19 chronic symptoms: a postinfectious entity? Clin Microbiol Infect. 2020;26:1448‐1449. 32712242 10.1016/j.cmi.2020.07.028 PMC7376333

[15] Nath A . Long‐haul COVID. Neurology. 2020;95:559‐560. 32788251 10.1212/WNL.0000000000010640

[16] Rubin R . As their numbers grow, COVID‐19 "Long Haulers" stump experts. JAMA. 2020;324(14):1381‐1383. 10.1001/jama.2020.17709 32965460

[17] Czeisler MÉ , Lane RI , Petrosky E , et al. Mental health, substance use, and suicidal ideation during the COVID‐19 pandemic ‐ United States, June 24–30, 2020. MMWR Morb Mortal Wkly Rep. 2020;69:1049‐1057. 32790653 10.15585/mmwr.mm6932a1 PMC7440121

